# Genome-wide identification and co-expression network analysis of *Aux/IAA* gene family in *Salvia miltiorrhiza*

**DOI:** 10.7717/peerj.15212

**Published:** 2023-04-17

**Authors:** Bin Huang, Yuxin Qi, Xueshuang Huang, Peng Yang

**Affiliations:** Hunan Provincial Key Laboratory for Synthetic Biology of Traditional Chinese Medicine, School of Pharmaceutical Sciences, Hunan University of Medicine, Huaihua, Hunan, China

**Keywords:** Aux/IAA, *Salvia miltiorrhiza*, WGCNA, regulation

## Abstract

The auxin/indole-3-acetic acid (Aux/IAA) gene family serves as a principal group of genes responsible for modulating plant growth and development through the auxin signaling pathway. Despite the significance of this gene family, the identification and characterization of members within the well-known Chinese medicinal herb *Salvia miltiorrhiza* (*S. miltiorrhiza*) have not been thoroughly investigated. In this study, we employed bioinformatics methods to identify 23 Aux/IAA genes within the genome of *S. miltiorrhiza*. These genes were classified into typical IAA and atypical IAA based on their domain structure. Our analysis of the promoter regions revealed that the expression of these genes is regulated not only by auxins, but also by other hormones and environmental factors. Furthermore, we found that the expression patterns of these genes varied across various tissues of *S. miltiorrhiza*. While our initial hypothesis suggested that the primary function of these genes was the interaction between *SmIAA* and *ARF*, gene co-expression network analysis revealed that they are also influenced by various other transcription factors, such as *WRKY* and *ERF*. The findings establish a sturdy basis for future investigations into the function of the Aux/IAA gene family and exhibit promising prospects for enhancing the genetics of this medicinal flora and its associated species.

## Introduction

Auxin, the first identified plant hormone, is present ubiquitously in all plants and participates in every stage of plant growth and development (Guilfoyle & Hagen, 2007). Indole-3-acetic acid (IAA) is a common plant auxin (Luo, Zhou & Zhang, 2018; Wu et al., 2017) and plays a crucial role in many processes of plant growth and development, such as cell division, embryogenesis, morphogenesis, directional response, dormancy extension, apical dominance, and tissue differentiation (Kepinski & Leyser, 2005; Leyser et al., 1996; Leyser, 2017; Luo, Zhou & Zhang, 2018; Mironova et al., 2017).

The response and signal transduction of plant cells to auxin are primarily mediated through the TIR1/AFB-Aux/IAA-ARF signaling pathway in the nucleus (Guilfoyle & Hagen, 2007). In this signaling pathway, auxin signal transduction is primarily controlled by auxin/indole-3-acetic acid (Aux/IAA) and auxin response factor (ARF) (Lavy & Estelle, 2016). In an environment with low concentrations of auxin, ARF and Aux/IAA protein form a heterodimer and bind to the TOPLESS (TPL) protein and histone deacetylase (HDACs) to inhibit the response of ARF to auxin (Luo, Zhou & Zhang, 2018; Parry et al., 2009; Winkler et al., 2017). However, when the concentration of auxin is high in the environment, auxin binds to the TIR1 receptor, and the resulting SCF^TIR1^ complex breaks down the dimer formed by Aux/IAA protein and ARF protein through ubiquitin (Szemenyei, Hannon & Long, 2008; Winkler et al., 2017). At the same time, proteases degrade Aux/IAA protein to restore ARF’s activity (Luo, Zhou & Zhang, 2018; Parry et al., 2009; Winkler et al., 2017).

Aux/IAA protein is a crucial gene involved in the early response to auxin, and plays a vital role in regulating plant growth and development (Luo, Zhou & Zhang, 2018). The protein typically consists of four distinct domains, as described in previous studies (Dreher et al., 2006; Guilfoyle & Hagen, 2007). Domain I, acting as a transcriptional repressor, can be repressed by the adjacent promoter region. It contains an EAR motif associated with the ethylene response factor (ERF) (Szemenyei, Hannon & Long, 2008; Tiwari, Hagen & Guilfoyle, 2003). The LxLxL motif in the EAR motif of domain I may attract TPLs, which, when combined with the conserved N-terminal TPD domain of TPLs, create a common repressor, thus serving as transcriptional suppressors of downstream auxin regulating genes. Domain II serves as the degradation determinant, featuring the conserved TIR1/AFB recognition sequence GWPPV, which plays a vital role in the stability of the Aux/IAA protein (Shimizu-Mitao & Kakimoto, 2014). The GWPPV motif is a combination of five amino acids, namely Gly-Trp-Pro-Pro-Val. Mutations in the GWPPV recognition sequence may delay the degradation of Aux/IAA and disrupt the auxin signal transduction pathway (Shimizu-Mitao & Kakimoto, 2014). Domain III, with its βαα-fold structure, bears structural and functional resemblance to the DNA recognition motif in Arc and Met J repressors (Dreher et al., 2006; Liu et al., 2011; Luo, Zhou & Zhang, 2018). The fourth and final domain, Domain IV, contains one acidic domain and one SV40 type NLS (PKKKRKV) domain (Dreher et al., 2006; Liu et al., 2011; Luo, Zhou & Zhang, 2018). Domains III and IV are identical to the C-terminal dimerization domain (CTD domain) of ARF proteins, capable of generating type I/II PB1 domains that interact with Aux/IAA and ARF family proteins to regulate ARF activity (Guilfoyle & Hagen, 2007; Luo, Zhou & Zhang, 2018; Winkler et al., 2017).

Currently, through genome-wide analysis, the Aux/IAA gene family has been identified in over 30 plant species. For example, *Arabidopsis thaliana* (*A. thaliana*) contains 29 IAA proteins (Liscum & Reed, 2002), *Oryza sativa* (*O. sativa*) contains 31 members (Jain et al., 2006), *Solanum tuberosum* (*S. tuberosum*) and *Vitis vinifera* (*V. vinifera*) all contain 26 members (Çakir, Kiliçkaya & Olcay, 2013; Gao et al., 2016). Certain Aux/IAA genes have been shown to play a crucial role in plant growth, among which the AtIAA7 protein may inhibit flowering time under short-day light by negatively regulating the expression of *GA20ox1* and *GA20ox2* genes (Luo, Zhou & Zhang, 2018; Mai, Wang & Yang, 2011). The AtIAA8 protein interacts with the AtARF6/8 protein and modulates the level of jasmonic acid (JA) to influence flower organ development (Wang et al., 2013). The AtIAA17 protein plays an important role in regulating leaf senescence (Shi et al., 2015). Functional analysis of the *AtIAA28* mutant revealed that *AtIAA28* promotes the transcription of lateral root initiation genes in response to auxin signals (Rogg, Lasswell & Bartel, 2001).

*Salvia miltiorrhiza* (*S. miltiorrhiza*), a member of the genus *Salvia* within the *Labiatae* family, is well known for its dried roots and rhizomes, which serve as a traditional Chinese medicine (Xu et al., 2016). Due to its advantageous growth characteristics, including a simple growth environment, short reproductive cycle, small genome size, and ease of tissue culture, *S. miltiorrhiza* has been established as a model system for the study of medicinal plants (Wang et al., 2021; Xu et al., 2016). The Aux/IAA gene family plays a crucial role in regulating plant growth and development (Guilfoyle & Hagen, 2007), making the identification of these genes in *S. miltiorrhiza* of paramount importance for understanding the developmental process and cellular response of auxin in this medicinal plant. This information could have important implications for the development of new plant varieties with improved growth and stress tolerance, as well as for the utilization of *S. miltiorrhiza* as a valuable medicinal resource. However, in *S. miltiorrhiza*, the use of the whole genome to study the Aux/IAA gene family has not been reported.

In the present investigation, we executed a comprehensive analysis of the Aux/IAA gene family in *S. miltiorrhiza* through a genome-wide approach. As a result, we successfully identified a total of 23 members of the *S. miltiorrhiza* Aux/IAA gene family (designated as *SmIAA*). Subsequently, we performed bioinformatic analyses to evaluate the physicochemical properties and sequence features of these genes. In addition, we utilized RNA-seq data to investigate the tissue-specific expression profiles of the *SmIAAs*, and further established a co-expression network to uncover the co-expression relationships between the *SmIAAs* and *transcription factors*.

## Materials and Methods

### Identification and characterization of the Aux/IAA gene family in *S. miltiorrhiza*

The Aux/IAA protein sequences of *A. thaliana* and *O. sativa* were downloaded from The Arabidopsis Information Resources (TAIR) database and Rice Genome Annotation Project (RGAP) database respectively. The genomic data of *S. miltiorrhiza* was downloaded from National Genomics Data Center (NGDC) database (project number GWHAOSJ00000000).

We employed two methods to identify potential members of the *SmIAAs*. First, we utilized the *A. thaliana* Aux/IAA protein sequence as a seed sequence for BLASTP homologous alignment in the *S. miltiorrhiza* local database. We then validated the domain structure of the resulting sequences using the Pfam database (El-Gebali et al., 2018) and retained only those sequences that contained the AUX_IAA domain as candidate members of the *SmIAAs*. Second, we utilized the iTAK database to comprehensively evaluate the entire *S. miltiorrhiza* protein sequence and identified additional members of the candidate *SmIAAs* (Zheng et al., 2016). By combining the candidate gene family members obtained by these two methods, we determined the final set of members comprising the *SmIAAs*.

To explore the evolutionary relationships among *SmIAAs*, we utilized the Neighbor-Joining (NJ) approach implemented in MEGA X software (Kumar et al., 2018), employing the following parameters: 50% partial deletion and 500 bootstrap replications. Additionally, we analyzed the basic physicochemical properties of the protein sequences using the Expasy online website (http://web.expasy.org/protparam/) and predicted their subcellular localization using the WoLF PSORT software (https://wolfpsort.hgc.jp/). To further investigate the chromosomal distribution of SmIAAs, we obtained relevant information from the *S. miltiorrhiza* genome annotation file and visualized it using the MG2C software (http://mg2c.iask.in/mg2c_v2.0/).

### Genetic analysis of Aux/IAA gene sequence and promoter cis-acting elements in *S. miltiorrhiza*

The structure of *SmIAA* gene sequence was analyzed by TBtools software according to the GFF3 file and CDS file of *S. miltiorrhiza* (Chen et al., 2020). The protein sequence domain of SmIAA proteins were mined by CD-search online database (https://www.ncbi.nlm.nih.gov/Structure/bwrpsb/bwrpsb.cgi). Finally, Tbtools software was used to visually analyze the results (Chen et al., 2020). Furthermore, GO enrichment analysis was performed using R software. In addition, the promoter sequence of 2,000 bp upstream of the start codon of the *SmIAAs* was extracted from the *S. miltiorrhiza* genome. Subsequently, the promoter cis-acting elements were analyzed using the online website PlantCARE (http://bioinformatics.psb.ugent.be/webtools/plantcare/html/), with the results of this analysis being visualized through TBtools software, as detailed in Table S1.

### Comparative analysis of Aux/IAA gene family across different species

In order to explore the genetic and evolutionary relationships among members of the Aux/IAA gene family in various species, a multiple sequence alignment of 23 SmIAA proteins, 29 AtIAA proteins, and 31 OsIAA proteins was performed using the online program Clustal Omega, followed by MEGAX to create the phylogenetic tree (Table S2), with the parameters used in line with previous analyses (Kumar et al., 2018; Sievers & Higgins, 2014). Furthermore, we utilized the Maximum Likelihood (ML) method to construct an additional evolutionary tree to compare with the results obtained using the NJ method (Fig. S4). This was done with the implementation of specific parameters, including a 50% partial deletion criterion and 500 bootstrap replications. The phylogenetic tree was shown using the online software Evolview (https://www.evolgenius.info/evolview/) (Subramanian et al., 2019). MEME, an online program (https://meme-suite.org/meme/), was used to assess conserved motifs.

### Collinearity analysis and selection pressure analysis

The GFF annotation file of the genome was utilized to acquire the chromosomal location data for *SmIAAs*. The MCScanX software, developed by Wang et al. (2012), was subsequently employed to compute the collinear block and tandem repeat gene information. The TBtools software (Chen et al., 2020) was utilized to visualize the chromosomal distribution and collinear relationships of the *SmIAAs*. For each identified homologous gene pair by MCScanX, the synonymous substitution rate (Ks) and non-synonymous substitution rate (Ka) were computed using KaKs_Calculator software (Wang et al., 2010) for each synonymous site. The Ka/Ks ratio was utilized to evaluate the rate of protein evolution and determine the selection pressure of the genes during evolution.

### RNA-seq analysis of *SmIAA* expression in *S. miltiorrhiza*

The RNA-seq data of *S. miltiorrhiza* were obtained from the NCBI database (PRJNA744957& PRJNA798876). The received sequence read archive (SRA) files were first converted to the fastq format using the fastq-dump program. The downloaded raw data was then trimmed and preprocessed using TrimGalore (https://github.com/FelixKrueger/TrimGalore), and the resulting clean data was utilized for downstream analysis. To construct a genome index with default settings, the Hisat2-build command in Hisat2 (version 2.3.0) (Zhang et al., 2021) was used. The featureCounts software was then employed to calculate the raw counts of each gene in each sample. The standardized transcriptional abundance was determined by computing the transcripts per million (TPM) value. The raw counts of each sample were used to generate a gene expression matrix of 21 transcriptome samples (Table S3). Finally, the expression data for the *SmIAAs* was extracted and a corresponding heat map was generated using the TBtools software (Chen et al., 2020).

### Gene co-expression network analysis in *S. miltiorrhiza*

Transcription factors were identified in the entire protein sequence of *Salvia miltiorrhiza* using the iTAK online software developed by Zheng et al. (2016). Gene co-expression network analysis was conducted using R software version 3.9.4 and the Weighted Gene Co-expression Network Analysis (WGCNA) package developed by Langfelder & Horvath (2008). First, the goodSamplesGenes function within the WGCNA package was utilized to identify and filter out any genes with missing values. The soft threshold power of 14 was then selected to construct the weighted gene network, with parameter settings of minModuleSize = 30, mergeCutHeight = 0.25, and maxBlockSize = 6,000. Additionally, to ensure the biological significance of each co-expression module, GO enrichment analysis was performed for each module using R software.

### Gene expression analysis of *S. miltiorrhiza* using qPCR and transcriptomics data integration

Two-year-old *S. miltiorrhiza* seedlings were collected and frozen in a −80 °C refrigerator. The roots, stems, leaves, and flowers were used for specific expression detection. Fluorescent quantitative specific primers (Table S4) were designed using Primer 5.0, and *SmUBQ* was used as an internal reference gene. Total RNA was extracted using the RNAprep Pure Plant Plus Kit (Tiangen, Beijing) and reverse transcribed using the PrimeScriptTM reverse transcriptase kit (Takara Tokyo, Japan). Real-time PCR reactions were performed using the PerfectStart Green qPCR SuperMix Kit (Transgene, Beijing), and fluorescence signals were collected using a qTOWER 3G PCR instrument. Each sample was analyzed in triplicate, and relative expression was calculated using the 2^−ΔΔCT^ method (Table S5). The correlation between transcriptome data and qPCR data was calculated and visualized by Sangerbox website (http://sangerbox.com/) (Shen et al., 2022).

## Result

### Identification of *Aux/IAA* gene family members in *S. miltiorrhiza*

In this study, two distinct methods were employed to identify the Aux/IAA gene family across the entire genome of *S. miltiorrhiza*. The initial approach involved using *Arabidopsis* Aux/IAA protein sequences to conduct a BLASTp search on the proteomic data of *S. miltiorrhiza*. Subsequently, the results were subjected to domain detection *via* submission to the Pfam database (El-Gebali et al., 2018), and the undesired outcomes were removed. This led to the identification of a total of 23 potential Aux/IAA gene family members. The second method involved employing the iTAK database to analyze the complete protein sequences of *S. miltiorrhiza*, which also resulted in the identification of 23 candidate genes. By comparing the outcomes of the two methods, it was established that the *S. miltiorrhiza* genome contained 23 members of the Aux/IAA gene family. In order to simplify future research, the nomenclature of *Arabidopsis genes* was adopted, and the genes were labeled as SmIAA1-SmIAA23 based on their distribution across the evolutionary tree (Fig. S1).

A comprehensive analysis was conducted to investigate the physicochemical properties of the SmIAA protein sequence. The results revealed that the number of amino acids in the SmIAA protein ranged from 87 to 371 aa, with an average of 211.61 aa. The molecular weight of the SmIAA protein was found to be between 10,364.81 and 40,197.45 Da, while the theoretical isoelectric point ranged from 4.82 to 9.81. Moreover, the subcellular localization analysis indicated that some proteins, namely SmIAA1, SmIAA4, SmIAA5, SmIAA8, SmIAA10, SmIAA11, SmIAA14, SmIAA16, SmIAA18, SmIAA22, and SmIAA23, were located in the cytoplasm, whereas the remaining proteins were all localized in the nucleus (Table 1).

**Table 1:** The physiological and biochemical features of SmIAAs. The Aux/IAA gene family in *S. miltiorrhiza*: the number of members, molecular weight, isoelectric point, and the presence of conserved domains.

Name	Gene ID	Accession ID	Chromosome localization			Mw (Da)	pI	CDS (bp)	Length (aa)	Subcellular localization	Homologs in Arabidopsis
SmIAA1	EVM0000296.1	GWHTAOSJ027615	Chr8	5920653	5921878	21688.8	9.81	573	190	Cytoplasm	AT3G62100
SmIAA2	EVM0009395.1	GWHTAOSJ003786	Chr1	57848442	57849325	20565.38	8.58	540	179	Nucleus	AT1G04550
SmIAA3	EVM0004349.1	GWHTAOSJ017483	Chr5	872484	876005	24827.71	5.94	657	218	Nucleus	AT3G62100
SmIAA4	EVM0022630.1	GWHTAOSJ006473	Chr2	327881	328425	10364.81	4.82	264	87	Cytoplasm	AT1G04550
SmIAA5	EVM0009745.1	GWHTAOSJ005170	Chr1	73323215	73324050	16049.36	9.25	420	139	Cytoplasm	AT5G57420
SmIAA6	EVM0021654.1	GWHTAOSJ023551	Chr6	60358018	60360065	20499.12	5.16	537	178	Nucleus	AT1G04550
SmIAA7	EVM0001033.1	GWHTAOSJ007657	Chr2	13722326	13725721	40197.45	9.38	1116	371	Nucleus	AT2G46990
SmIAA8	EVM0026407.1	GWHTAOSJ019954	Chr5	58348218	58351945	28501.2	8.64	810	269	Cytoplasm	AT2G33310
SmIAA9	EVM0002334.1	GWHTAOSJ016330	Chr4	66298635	66301163	34131.41	9	927	308	Nucleus	AT5G25890
SmIAA10	EVM0010171.1	GWHTAOSJ019616	Chr5	55028101	55031170	18580.66	8.17	498	165	Cytoplasm	AT1G15580
SmIAA11	EVM0027652.1	GWHTAOSJ019676	Chr5	55711914	55716112	19848.71	5.41	534	177	Cytoplasm	AT1G15580
SmIAA12	EVM0010443.1	GWHTAOSJ015659	Chr4	57556179	57562422	38312.09	7.6	1077	358	Nucleus	AT3G23050
SmIAA13	EVM0025242.1	GWHTAOSJ011536	Chr3	21196572	21199823	36647.47	8.6	1023	340	Nucleus	AT3G04730
SmIAA14	EVM0003584.1	GWHTAOSJ019677	Chr5	55738962	55740433	21585.91	8.15	594	197	Cytoplasm	AT1G80390
SmIAA15	EVM0019034.1	GWHTAOSJ019617	Chr5	55050465	55052967	20718.73	7.72	573	190	Nucleus	AT5G43700
SmIAA16	EVM0009041.1	GWHTAOSJ007792	Chr2	15980426	15981800	20799.85	6.73	561	186	Cytoplasm	AT4G14550
SmIAA17	EVM0020724.1	GWHTAOSJ007565	Chr2	12458073	12462291	26535.11	8.96	735	244	Nucleus	AT3G04730
SmIAA18	EVM0011993.1	GWHTAOSJ020429	Chr5	62919943	62922717	24660.27	6.02	687	228	Cytoplasm	AT4G14550
SmIAA19	EVM0021151.1	GWHTAOSJ010857	Chr3	12283059	12284558	25314.25	7.54	684	227	Nucleus	AT4G14550
SmIAA20	EVM0010879.1	GWHTAOSJ020426	Chr5	62894169	62895673	17843.23	5.31	489	162	Nucleus	AT5G43700
SmIAA21	EVM0004282.1	GWHTAOSJ004659	Chr1	68141757	68142829	20661.38	6.61	561	186	Nucleus	AT5G43700
SmIAA22	EVM0006475.1	GWHTAOSJ007793	Chr2	16036879	16038173	19453.26	5.39	519	172	Cytoplasm	AT5G43700
SmIAA23	EVM0007611.1	GWHTAOSJ000893	contig685	54663	55050	10916.64	5.09	291	96	Cytoplasm	AT4G14560

Furthermore, we referred to the genome annotation information of *S. miltiorrhiza* to identify the distribution patterns of *SmIAAs*, with the exception of *SmIAA23* on contig685. Our analysis revealed that these genes were situated on chromosomes other than chromosome 7, with most genes located at the upper and lower ends of the chromosome. The highest frequency of gene distribution was observed on chromosome 5 (eight genes), while the lowest was on chromosomes 6 and 8 (one gene). Moreover, the genes on chromosomes 2 and 5 were more densely distributed compared to those on other chromosomes, which were more sparsely and randomly distributed (Fig. S2).

### Analysis of gene structure and protein sequence

**Figure 1:**
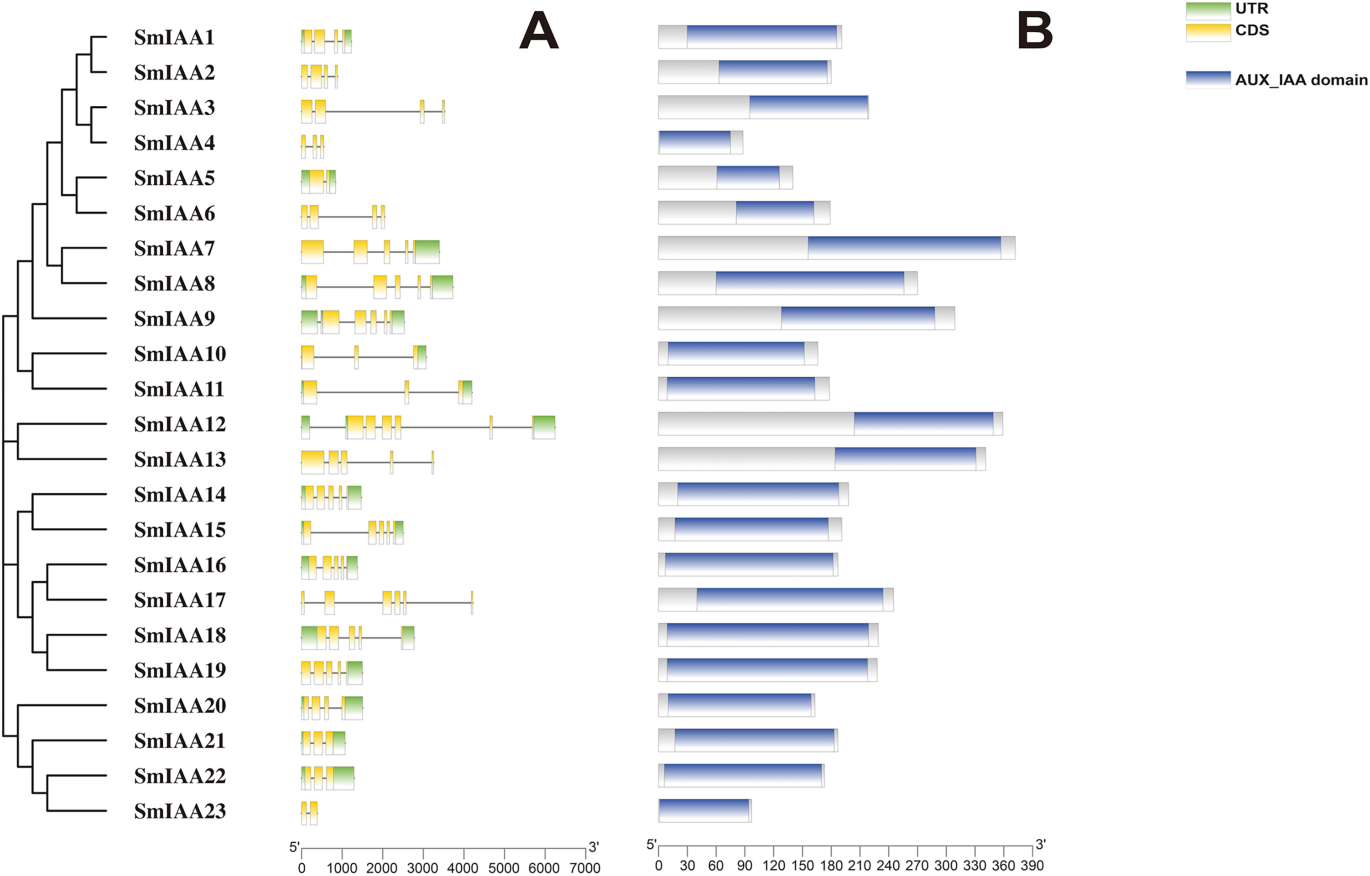
The gene structure and protein structure analysis of *SmIAAs*. (A) The upper panel shows the gene structure of the *SmIAAs*. The CDS regions are represented by yellow boxes, respectively. The untranslated regions (UTRs) are represented by green boxes. The scale bar indicates the length of the gene structure in base pairs. (B) The protein structures of SmIAA proteins were predicted using the Pfam database. The AUX_IAA domain contained in SmIAA proteins is represented by a graded blue box. The evolutionary tree was constructed using the neighbor-joining (NJ) method, based on the multiple sequence alignment of SmIAA protein sequences. The evolutionary distances were calculated using the Poisson correction method. The whole figure was created using Tbtools, a software tool for the visualization and annotation of biological data.

Previous studies have demonstrated that gene structural diversity is a significant driver for the evolution of many gene families (Singh & Jain, 2015). To further explore the gene structure of *SmIAAs* (Fig. 1A), we conducted an analysis which revealed variable splicing in all genes. The number of introns ranged from 1 to 6. Notably, some genes did not have UTRs. *SmIAA2, SmIAA3, SmIAA4, SmIAA6, SmIAA13, SmIAA17*, and *SmIAA23* contained only CDS and no UTR.

**Figure 2:**
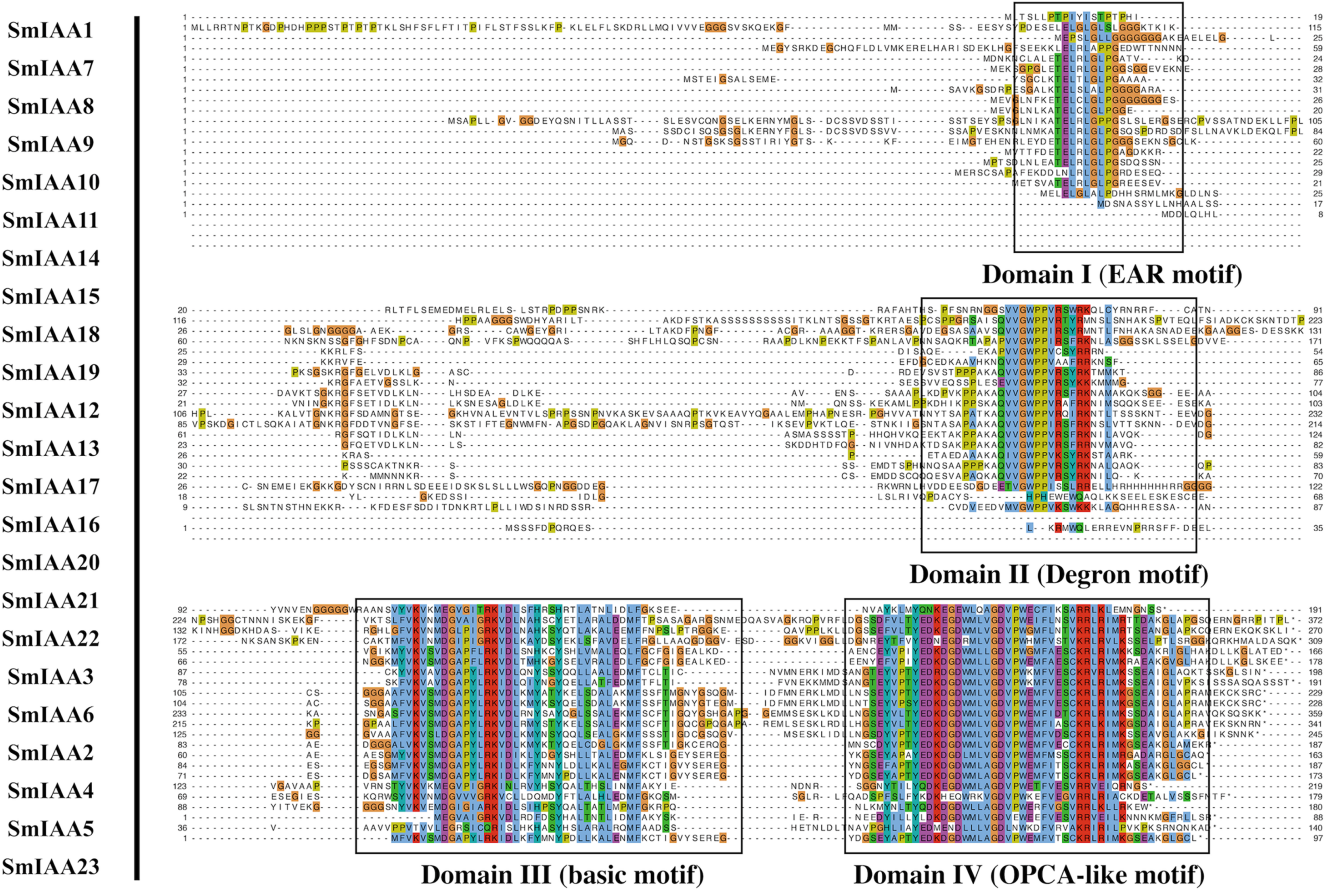
Multiple sequence alignment analysis of SmIAA family proteins. The figure shows the results of a multiple sequence alignment analysis of SmIAA proteins. The alignment was performed using the Clustal Omega program, and the results were visualized using the Jalview program. The different regions of the proteins are color-coded, with the conserved domains indicated by different colored boxes. The numbers above the alignment indicate the position of each residue in the protein sequence. The alignment reveals that the SmIAA proteins share a high degree of sequence conservation in the conserved domains, including the AUX/IAA domain and the domain II/III. The variable regions of the proteins are less conserved, reflecting their divergent functions. The alignment also reveals the presence of several conserved motifs, which may be involved in protein-protein interactions or other functions.

To gain more insights into the properties of SmIAA proteins, we performed domain analysis using the Pfam database. The results showed that all proteins contained Aux/IAA domains, although their placement and size varied (Fig. 1B). We also conducted multiple sequence alignment to evaluate the conserved amino acid sites in the SmIAA proteins. The domains of SmIAA proteins were further divided into Domain I (EAR-motif), Domain II (Degron motif), Domain III (Basic motif), and Domain IV (OPCA-like motif) (Figs. 2 and S3). Some SmIAA proteins were found to be atypical Aux/IAA proteins, lacking at least one conserved domain. This structural diversity may provide these proteins with diverse functions, although further research is required.

### Phylogenetic analysis of SmIAA proteins

**Figure 3:**
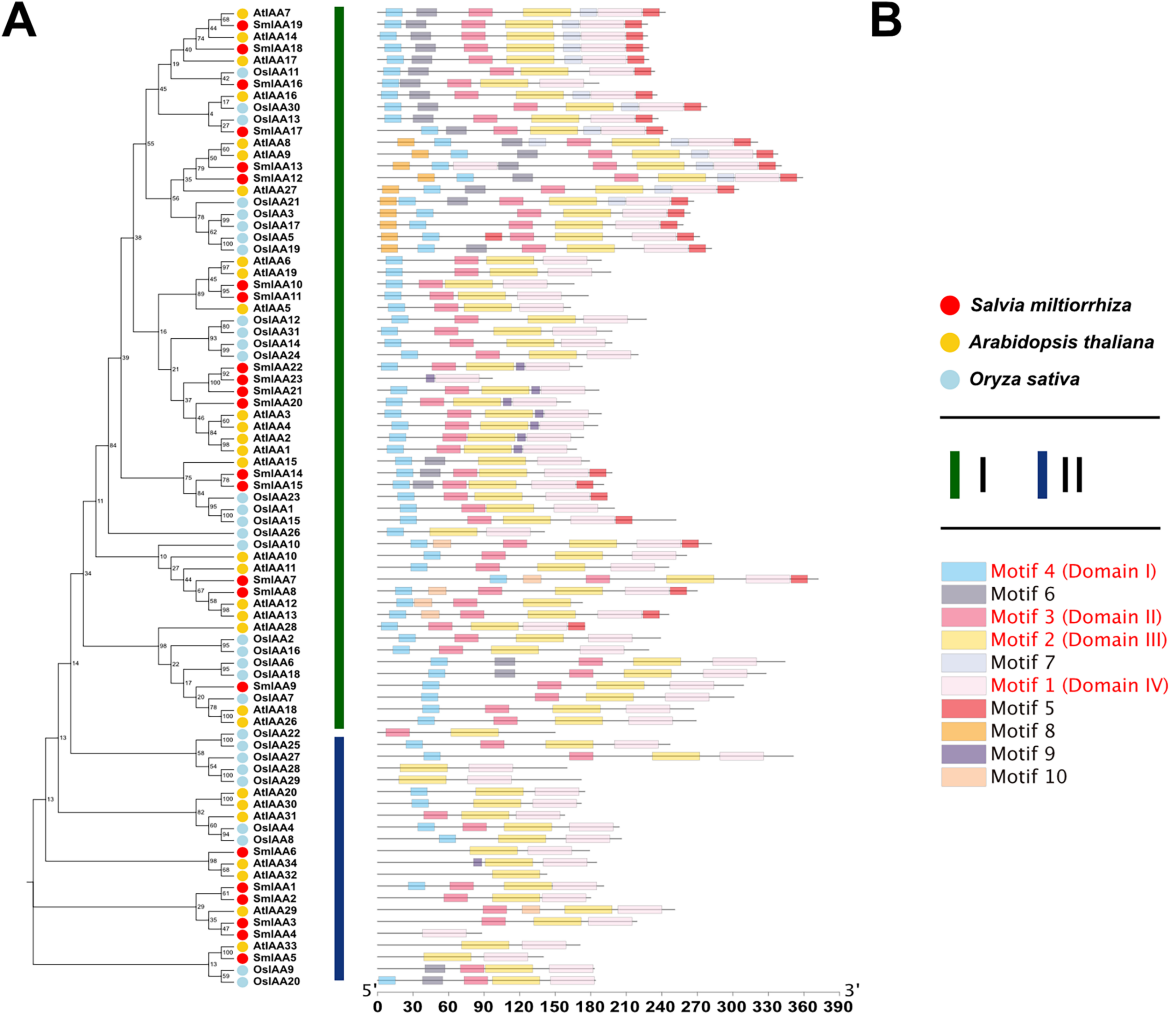
Phylogenetic tree and conserved motif analysis of Aux/IAA family genes in *A. thaliana, O. sativa, and S. miltiorrhiza*. (A) The evolutionary tree was constructed using the neighbor-joining (NJ) method, based on the multiple sequence alignment of Aux/IAA family protein sequences from *Arabidopsis thaliana*, *Oryza sativa*, and *Salvia miltiorrhiza*. The evolutionary distances were calculated using the Poisson correction method, and the numbers on the branches represent the bootstrap values (in percentage) from 500 replicates. The different clades are labeled with different colors, corresponding to the different species shown in the figure. All the sequences used to construct the evolutionary tree can be obtained from Table S2. (B) The conserved motifs of the Aux/IAA proteins were identified using the MEME Suite software. The different motifs are represented by different colored boxes. The motifs that are specially marked are related to the unique domain of Aux/IAA.

To investigate the potential relationship between typical and atypical AUX/IAA proteins, we identified and selected the Aux/IAA proteins from two model organisms, *A. thaliana* and *O. sativa*, as well as *S. miltiorrhiza* for further study. We utilized MEGA X to construct phylogenetic trees of the Aux/IAA proteins in three species. The results obtained from the ML method and NJ method were largely consistent, indicating the reliability of our findings. Additionally, we employed the MEME database to analyze the motifs present in these proteins. The resulting evolutionary tree was primarily divided into two groups, I and II, respectively. As illustrated in Fig. 3, the evolutionary tree and conserved motifs were consistent with previous studies on the Aux/IAA family of plants. The majority of the members were classified in Group I, with nearly all of them being canonical Aux/IAA proteins possessing four canonical domains (motif1, 2, 3, and 4). On the other hand, nearly all the members of Group II were found to be atypical Aux/IAA proteins, lacking at least one conventional domain. Atypical Aux/IAA proteins have been found to be prevalent in the Aux/IAA gene family of various plants, where they play a significant role in plant adaptability to changing environmental conditions, although their specific functions remain largely unknown (Luo, Zhou & Zhang, 2018).

### Analysis of collinearity and selection pressure

**Figure 4:**
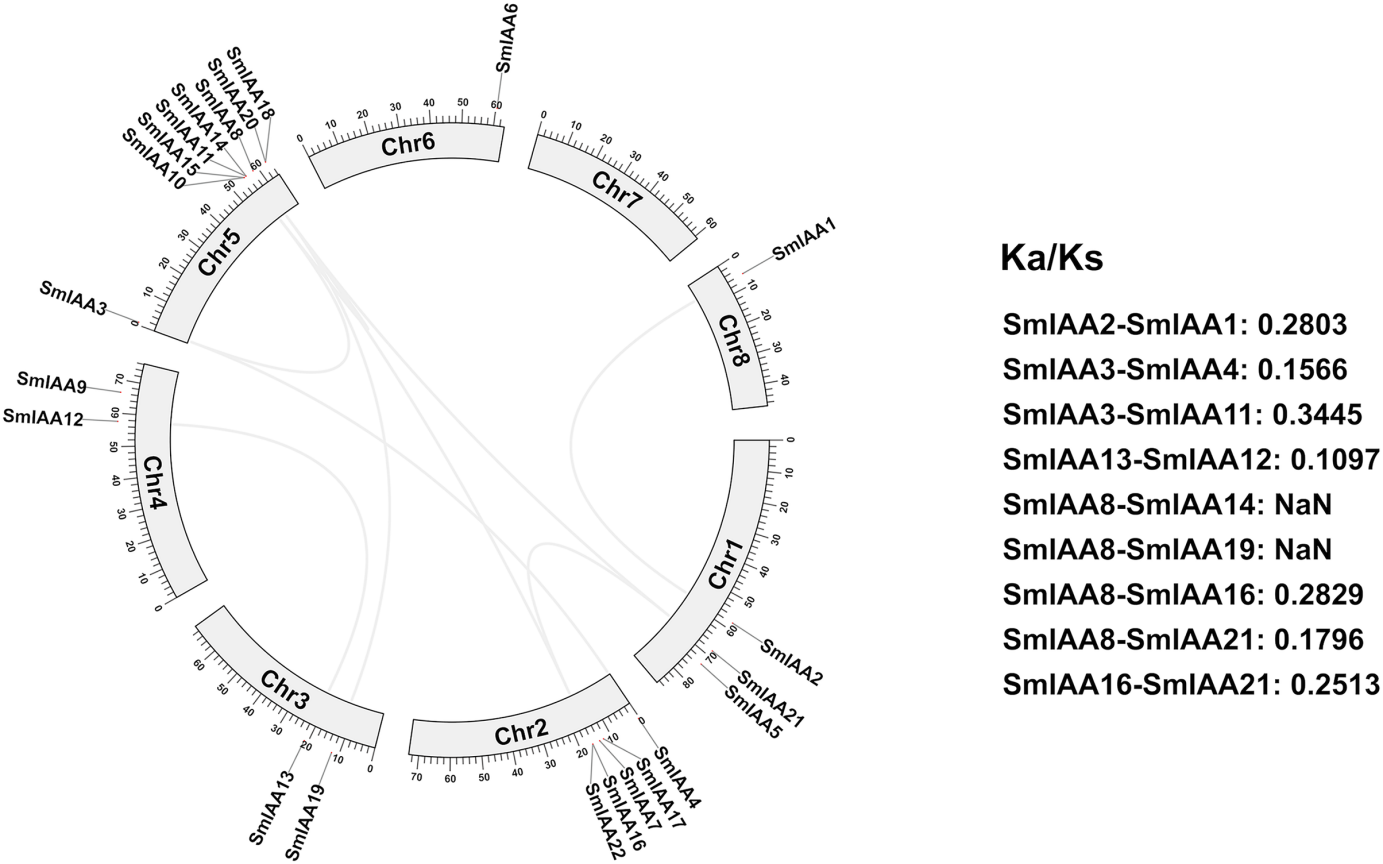
Collinearity and selection pressure analysis of *SmIAAs*. The collinearity analysis of *SmIAAs* was performed using the MCScanX software, and the results are shown as syntenic blocks. The lines connecting the genes represent the syntenic relationships between them. The collinearity analysis revealed several syntenic gene pairs, indicating the occurrence of gene duplication events during the evolution of *SmIAAs*. The Ka/Ks ratio analysis was used to measure the selection pressure acting on each syntenic gene pair. The Ka/Ks ratios were calculated using the KaKs_Calculator software.

We conducted a collinear analysis of *SmIAAs* to elucidate their relationships further. The results showed that nine lineal homologous gene pairs were identified among all *SmIAAs*, suggesting that chromosomal replication processes play a crucial role in the amplification of the *S. miltiorrhiza* Aux/IAA gene family members (Fig. 4).

To gain a better understanding of the evolutionary processes of genes and proteins, studying selective interaction patterns can be a useful approach. Selection has been shown to be a valuable technique for speculating about gene function (Wang et al., 2009). The Ka/Ks value, which is the ratio of the two protein-coding genes’ non-synonymous substitution rates (Ka) and synonymous substitution rates (Ks), can be used to identify whether the gene is under selection pressure. When Ka/Ks > 1, the gene is thought to be positively selected; when Ka/Ks = 1, the gene is thought to be purified and selected; and when Ka/Ks < 1, the gene is thought to be neutrally selected. Except for *SmIAA8/SmIAA14* and *SmIAA8/SmIAA19*, the Ka/Ks value of the other *S. miltiorrhiza* lineal homologous gene pairs is less than 1, which means that these *SmIAAs* evolved under purification selection, indicating that the sequence of the Aux/IAA gene is relatively conservative. These findings suggest that the Aux/IAA gene plays a significant role in *S. miltiorrhiza* and attains an optimal state, which is consistent with the conservative function of several *SmIAAs*.

### Analysis of GO enrichment and *cis*-acting element

The GO categorization system is a widely used method for characterizing genes and proteins in various organisms. The system is organized into three distinct categories, including molecular function, biological process, and cellular component, with each category corresponding to specific functions or properties. To investigate the potential functions of the *SmIAAs*, we utilized TBtools to examine the GO enrichment of *SmIAA* and identified the top 10 most significantly enriched GO terms across the three categories (if less than 10, all).

**Figure 5:**
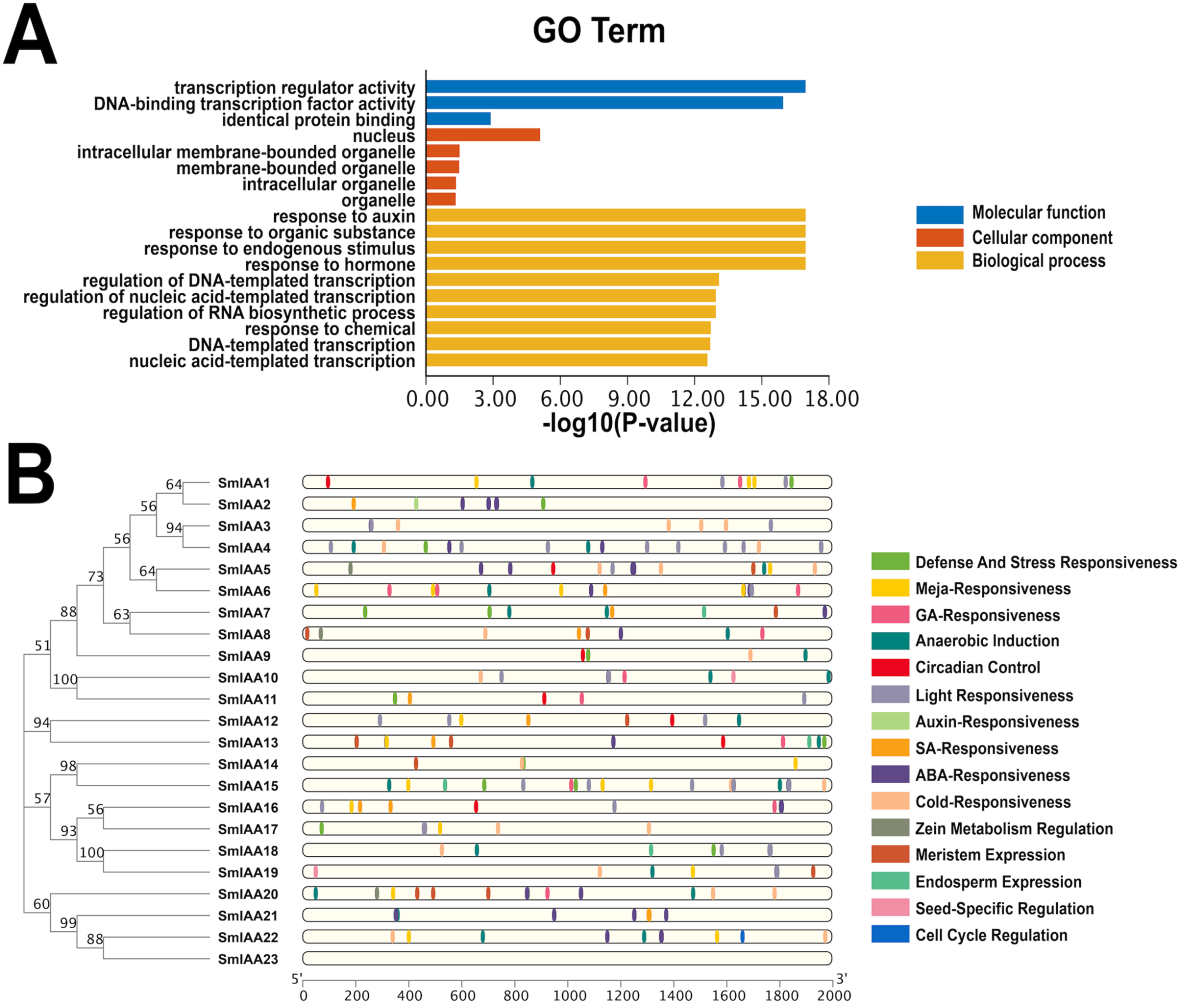
GO enrichment and various cis-element analysis of *SmIAAs*. (A) GO enrichment analysis was performed to investigate the biological functions of the *SmIAAs*. The GO terms are grouped into three main categories: biological process, cellular component, and molecular function. (B) The presence of various *cis*-elements in the promoter regions of the SmIAA genes was analyzed using the PlantCARE database. The different colored bars represent different *cis*-elements. The *cis*-elements are grouped into different categories, including hormone-related, stress-related, and development-related *cis*-elements.

Figure 5A demonstrates that the *SmIAAs* are associated with 10, five, and three GO terms related to biological process, cellular component, and molecular function, respectively. The majority of *SmIAAs* are believed to play a role in various biological activities. Their molecular functions include transcription regulator activity, DNA-binding transcription factor activity, and identical protein binding. The cell composition mainly includes the nucleus, nuclear, intracellular membrane-bounded organelle, membrane-bounded organelle, and other cellular components. Furthermore, *SmIAAs* are primarily involved in biological processes related to the response to auxin, organic substance, endogenous stimulus, and hormone, among others.

To gain a more comprehensive understanding of the role of *SmIAAs* in the growth and development of *S. miltiorrhiza*, we analyzed the cis-acting elements in the promoter region of the related genes, which is 2,000 bp upstream of the initiation codon (Fig. 5B and Table S1). Our analysis revealed the presence of numerous cis-acting elements associated with auxin, salicylic acid, abscisic acid, methyl jasmonate, and gibberellin in the promoter region of *SmIAAs*, in addition to key cis-acting elements associated with transcription and light response. The presence of these cis-regulatory components suggests that hormones have the capacity to regulate *SmIAAs*. Overall, our analysis of cis-acting elements reveals that different family members contain a variety of response motifs, implying that the SmIAA gene family may participate in various physiological processes of *S. miltiorrhiza* by responding to various response elements such as hormone, low temperature, light, and so on.

### Analysis of gene expression pattern

**Figure 6:**
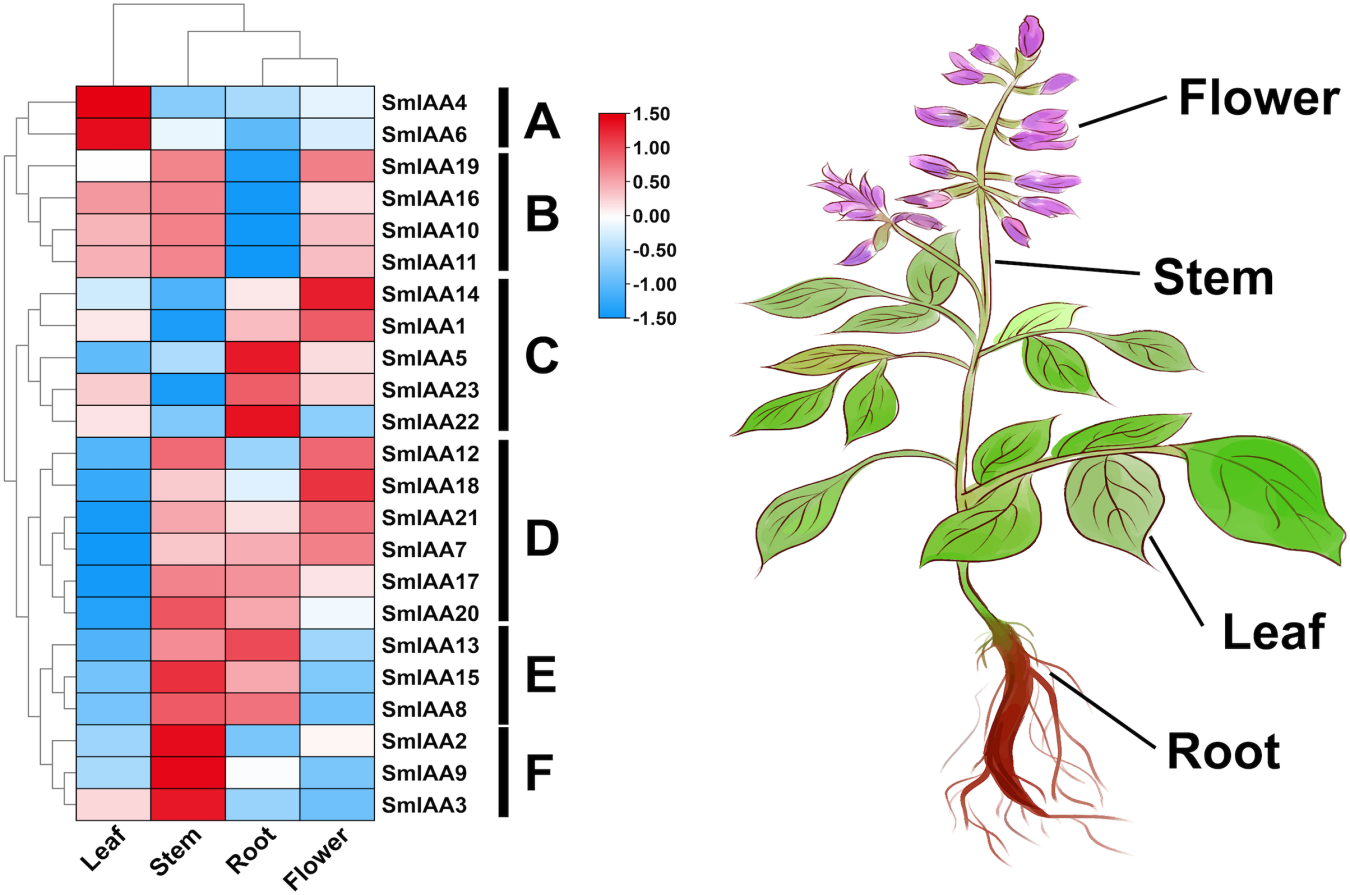
(A–F) Expression profiles of *SmIAAs* in different tissues of *S. miltiorrhiza*. The expression patterns of *SmIAAs* in different tissues of *S. miltiorrhiza* were analyzed using transcriptome data. Heat map visualization of the expression profiles was generated using TPM values, and row clustering was performed to group genes with similar expression patterns together. The heat map shows the expression levels of the *SmIAAs* across different tissues of *S. miltiorrhiza*, including root, stem, leaf, and flower. The color scale represents the relative expression level of each gene, with red indicating high expression and blue indicating low expression. The clustering of the genes reveals several groups with similar expression patterns, indicating potential functional similarities among these genes.

The temporal and spatial expression of a gene may provide insights into its functional features (Wang et al., 2021). In this study, transcriptome data retrieved from NCBI was used to extract the TPM values of root, stem, leaf, and flower tissues of *S. miltiorrhiza*, and subsequently analyze the expression pattern of the *SmIAAs*. The results of gene expression patterns based on transcriptome data indicated considerable variability in the expression of several *SmIAAs* across different tissues of *S. miltiorrhiza*, as well as in the expression trends of individual genes. Based on the results of expression cluster analysis, *SmIAAs* could be categorized into six groups (A~F) (Fig. 6). Group A consisted of two genes (*SmIAA4* and *SmIAA6*) that were primarily expressed in the leaves of *S. miltiorrhiza*. Group B included four genes (*SmIAA19, SmIAA16, SmIAA10*, and *SmIAA11*) that showed similar expression levels in the leaves, stems, and flowers of *S. miltiorrhiza*, and relatively low expression levels in the roots. Group C comprised five genes (*SmIAA14, SmIAA1, SmIAA5, SmIAA23*, and *SmIAA22*) that were predominantly expressed in the flowers and roots of *S. miltiorrhiza*. Group D encompassed six genes (*SmIAA12, SmIAA18, SmIAA21, SmIAA7, SmIAA17*, and *SmIAA20*) that showed the highest expression levels in the stems, roots, and flowers of *S. miltiorrhiza*, but minimal expression in the leaves. Group E consisted of three genes (*SmIAA13, SmIAA15*, and *SmIAA8*) that were primarily expressed in the stems and roots of *S. miltiorrhiza*. However, the three genes in group E (*SmIAA2, SmIAA9*, and *SmIAA3*) were significantly expressed only in the stem of *S. miltiorrhiza*. In summary, *SmIAAs* were expressed in clusters in various tissues of *S. miltiorrhiza*, with the majority of *SmIAAs* exhibiting high expression levels in roots, stems, and particularly flowers.

### Weighted correlation network analysis

**Figure 7:**
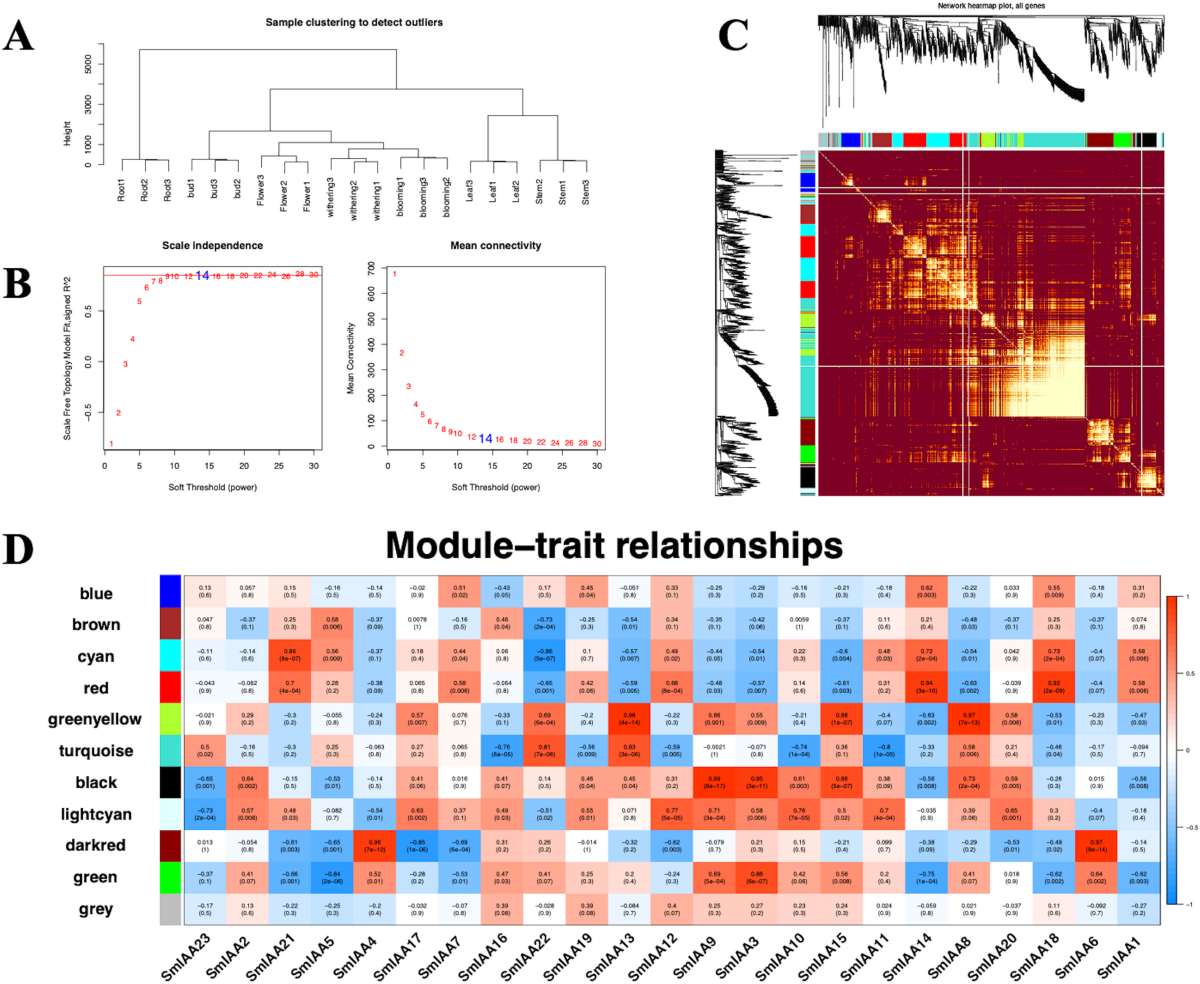
(A–D) The correlation diagram between the module and *SmIAAs* calculated by the WGCNA method. To investigate the correlation between SmIAA genes and different gene modules, a weighted gene co-expression network analysis (WGCNA) was performed based on transcriptome data from different tissues of *S. miltiorrhiza*.

Although the Aux/IAA gene family is crucial for auxin signal transduction, prior studies have suggested that its members may interact with other transcription factors (TFs) besides *ARFs* (Luo, Zhou & Zhang, 2018). To explore these interactions, we employed the iTAK tool to identify all TFs in the *S. miltiorrhiza* genome and utilized weighted correlation network analysis (WGCNA) to examine additional transcriptome data from *S. miltiorrhiza* flower tissue at different stages (withering, full blooming, and bud). Our analysis identified 68 TFs, including *ARF*, *ERF*, *MYB*, and others (Tables 2 and S6). We used a soft threshold of β = 14 to divide the 1814 TFs into 10 modules, with gene numbers ranging from 45 to 490 in each module (Table S7). The outcomes of the GO enrichment analysis of distinct modules revealed that each of these modules has distinct biological significance (Table S8). Furthermore, utilizing the Pearson correlation data, we observed a significant association between *SmIAAs* and the modules, suggesting that *SmIAAs* may be linked with the module hub genes (Fig. 7). We selected the red and cyan modules for further investigation, as they were highly associated with *SmIAA14* and negatively related to *SmIAA22*, respectively. We identified the top 10 hub genes in each module and annotated their homologs in the iTAK database (Table 3). Surprisingly, only one *ARF* was among the 20 key genes identified across various modules (EVM0019866), suggesting that *SmIAAs* may have other important functions beyond their role in the auxin pathway. However, more research is needed to confirm this hypothesis.

**Table 2:** Different transcription factors in the *S. miltiorrhiza* genome. The different transcription factors identified in the genome of *S. miltiorrhiza* using iTAK tool.

Total				1814			
Class	Num	Class	Num	Class	Num	Class	Num
Alfin-like	7	CAMTA	4	HB-other	12	OFP	25
AP2/ERF-AP2	19	CPP	8	HB-PHD	2	PLATZ	12
AP2/ERF-ERF	143	CSD	4	HB-WOX	15	RWP-RK	17
AP2/ERF-RAV	3	DBB	5	HRT	1	S1Fa-like	2
B3	44	DBP	2	HSF	34	SAP	2
B3-ARF	26	DDT	8	LFY	1	SBP	17
BBR-BPC	6	E2F-DP	8	LIM	10	SRS	9
BES1	9	EIL	7	LOB	50	STAT	1
bHLH	134	FAR1	43	MADS-M-type	25	TCP	29
bZIP	67	GARP-ARR-B	12	MADS-MIKC	46	Tify	11
C2C2-CO-like	14	GARP-G2-like	48	MYB	145	Trihelix	31
C2C2-Dof	34	GeBP	10	MYB-related	65	TUB	12
C2C2-GATA	28	GRAS	62	NAC	84	ULT	1
C2C2-LSD	5	GRF	12	NF-X1	3	VOZ	2
C2C2-YABBY	9	HB-BELL	15	NF-YA	8	Whirly	2
C2H2	110	HB-HD-ZIP	43	NF-YB	21	WRKY	78
C3H	56	HB-KNOX	11	NF-YC	9	zf-HD	16

**Table 3:** Top 10 hub genes in the black and cyan modules. The hub genes were identified using the weighted gene co-expression network analysis (WGCNA) method. The table includes information about the module color, gene name, gene annotation, correlation, *p*-value, and the K-MEANS-based connectivity (KME).

Group	Gene	Annotation	R	*P*	KME
Black	EVM0007532	WRKY	0.9931	3.72E−19	0.9931
Black	EVM0000041	bHLH	0.9929	5.06E−19	0.9929
Black	EVM0019585	zf-HD	0.9865	2.14E−16	0.9865
Black	EVM0012769	MYB	0.9857	3.78E−16	0.9857
Black	EVM0010344	bHLH	0.9851	5.29E−16	0.9851
Black	EVM0018729	HB-WOX	0.9840	1.05E−15	0.9840
Black	EVM0021887	AP2/ERF-ERF	0.9800	8.72E−15	0.9800
Black	EVM0023258	SBP	0.9797	9.96E−15	0.9797
Black	EVM0015830	zf-HD	0.9791	1.32E−14	0.9791
Black	EVM0011877	bHLH	0.9737	1.15E−13	0.9737
Cyan	EVM0017797	DBB	0.9906	7.35E−18	0.9906
Cyan	EVM0019866	B3-ARF	0.9763	4.30E−14	0.9763
Cyan	EVM0001762	C3H	0.9744	9.03E−14	0.9744
Cyan	EVM0004701	MYB	0.9731	1.45E−13	0.9731
Cyan	EVM0002416	HSF	0.9697	4.34E−13	0.9697
Cyan	EVM0011962	bZIP	0.9662	1.23E−12	0.9662
Cyan	EVM0004337	MYB-related	0.9645	1.91E−12	0.9645
Cyan	EVM0016656	C2H2	0.9607	5.02E−12	0.9607
Cyan	EVM0025053	MYB-related	0.9584	8.45E−12	0.9584
Cyan	EVM0000238	C2C2-Dof	0.9583	8.73E−12	0.9583

### The validation of qRT-PCR

**Figure 8:**
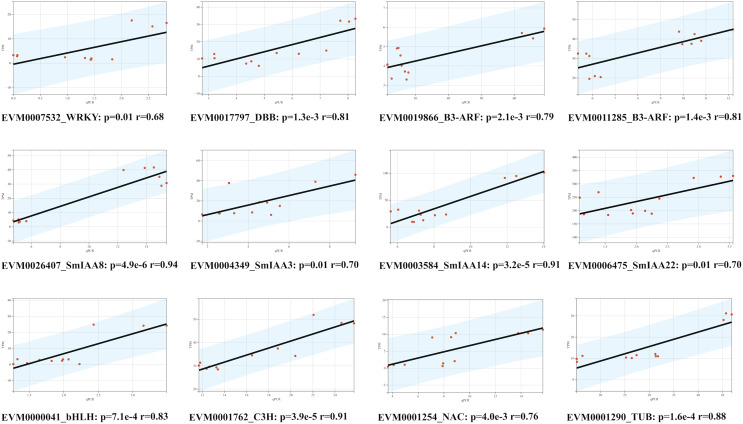
Correlation analysis between RNA-seq and qPCR data of 12 randomly selected *SmIAAs*. To validate the RNA-seq data, quantitative PCR (qPCR) was performed on 12 randomly selected *SmIAAs*. The expression levels of these genes were quantified by both RNA-seq and qPCR, and the correlation coefficient (R^2^) was calculated to compare the results. The results presented in this figure demonstrate a strong positive correlation (R^2^ > 0.8) between the RNA-seq and qPCR data for all 12 genes, indicating that the RNA-seq data is reliable and accurate.

To confirm the RNA-Seq results, we employed the quantitative reverse transcription polymerase chain reaction (qRT-PCR) technique to evaluate the expression levels of 12 genes, comprising of four *SmIAAs*, four hub genes selected by co-expression modules, and four transcription factors chosen randomly (Fig. 8). The findings demonstrated that the alteration in gene expression detected by qRT-PCR and RNA-Seq techniques was similar, and the outcomes of the two datasets were positively correlated, suggesting that the co-expression network established in this study is reliable.

## Discussion

The Aux/IAA gene family is a large and diverse family of transcriptional regulators that plays a critical role in the response pathway of plant auxin. The family has been identified and studied in a wide range of plant species, from soybean to wheat, tomato, and March plum (Gao et al., 2016; Jain et al., 2006; Liscum & Reed, 2002; Luo, Zhou & Zhang, 2018). The family members share a common structure that includes four conserved domains, and their expression is regulated by the presence and concentration of auxin in plant cells. This study focuses on the identification, structural, and phylogenetic analysis of the Aux/IAA gene family in *S. miltiorrhiza*. On the whole, the number of *SmIAAs* is similar to that of Aux/IAA gene family members in *A. thaliana* (Liscum & Reed, 2002). Most of the *SmIAAs* are located on chromosomes other than chromosome 7, and many of them are situated at the ends of the chromosomes. This suggests that the expansion of the *SmIAAs* may have been caused by tandem repeats.

Protein domains are important functional units, and conserved motifs are subunits of domains that contribute to the diverse biological functions of the domains. In the proteins encoded by the *SmIAAs*, 17 members contained four typical conserved domains of the family, while the remaining six members had deletions of these typical domains. These atypical Aux/IAA proteins are mainly missing Domain I (EAR-motif), so it is inferred that the transcriptional inhibitory function of these six members of *S. miltiorrhiza* on downstream ARF may be missing (Luo, Zhou & Zhang, 2018). It is reported that atypical Aux/IAA proteins are ubiquitous in various plant *Aux/IAA* gene families and play an important role in plant adaptation to changing environmental conditions, but more specific functions still need to be further studied (Li et al., 2017; Luo, Zhou & Zhang, 2018).

In addition to four typical conserved motifs, there are six conserved motifs in Aux/IAA protein. The overlap and difference of conserved motifs among different members reflect its functional characteristics to some extent. Other different conserved motifs may enable members to participate in functions other than auxin signal pathway, and the different expression patterns of members in different tissues of *S. miltiorrhiza* indicate that these different conserved motifs play different roles to a certain extent, but the specific functions of their effects need to be further studied. Moreover, the promoter analysis of *SmIAAs* suggests that they may respond to various stimuli, including auxin, gibberellin, abscisic acid, salicylic acid, methyl jasmonate, drought, salt, heat stress, and low temperature. The presence of these cis-acting elements may explain why *SmIAAs* have a wide range of functions in *S. miltiorrhiza*, including tissue-specific roles. However, further experimental research is required to confirm this. Overall, the physicochemical properties of SmIAA proteins contribute to their regulation of gene expression and involvement in plant adaptation to changing environmental conditions.

Transcriptome analysis showed that the expression of different Aux/IAA family genes was tissue specific, but Aux/IAA family genes were involved in each different tissue of *S. miltiorrhiza*. Previous reports have pointed out that *Aux/IAA* gene not only interacts with ARF, but also is regulated by other transcription factors (Guilfoyle & Hagen, 2007; Luo, Zhou & Zhang, 2018). In this study, co-expression network analysis was performed using transcriptome data from *S. miltiorrhiza* to identify transcription factors that may interact with the *SmIAAs*. This analysis identified 10 modules, and further correlation analysis revealed that each module was negatively correlated with at least one *SmIAA*. Analysis of the specific expression patterns of different *SmIAAs* in different tissues suggests that the different modules may play different roles in different tissues of *S. miltiorrhiza* (Table 4). Further analysis of the black and red modules revealed an interesting finding: although the Aux/IAA family is known to play an important role in auxin signaling, the hub genes in these two modules had only a small number of *ARFs* that were significantly associated with *SmIAAs*. This suggests that the *SmIAAs* may have additional, unknown functions in *S. miltiorrhiza*, but further research is needed to confirm this.

**Table 4:** The relationship between different modules and different tissues of *S*. *miltiorrhiza*. The modules were identified using the weighted gene co-expression network analysis (WGCNA) method. The table includes information about the module color, *SmIAAs* in each module, and the tissues in which the module was highly expressed.

Module	R	Gene	Tissue
Green	−0.62	SmIAA1	Flower
Darkred	−0.69	SmIAA7	Flower
Lightcyan	0.77	SmIAA12	Flower
Red	0.94	SmIAA14	Flower
Red	0.92	SmIAA18	Flower
Cyan	0.86	SmIAA21	Flower
Darkred	0.96	SmIAA4	Leaf
Darkred	0.97	SmIAA6	Leaf
Green	−0.84	SmIAA5	Root
Cyan	−0.86	SmIAA22	Root
Lightcyan	−0.73	SmIAA23	Root
Black	0.64	SmIAA2	Stem
Black	0.95	SmIAA3	Stem
Black	0.99	SmIAA9	Stem
Lightcyan	0.76	SmIAA10	Stem&Flower
Turquoise	−0.8	SmIAA11	Stem&Flower
Turquoise	−0.76	SmIAA16	Stem&Flower
Turquoise	−0.56	SmIAA19	Stem&Flower
Greenyellow	0.97	SmIAA8	Stem&Root
Greenyellow	0.98	SmIAA13	Stem&Root
Greenyellow	0.88	SmIAA15	Stem&Root
Darkred	−0.85	SmIAA17	Stem&Root
Lightcyan	0.65	SmIAA20	Stem&Root

## Conclusions

In this study, a comprehensive analysis was conducted on the Aux/IAA gene family in *S. miltiorrhiza*, which included whole genome identification, physicochemical property analysis, and expression analysis. Moreover, a gene co-expression network was constructed to investigate the interactions of *SmIAAs* with transcription factors. The findings revealed that this gene family comprised numerous members with diverse structures and functions. Notably, it was observed that these genes are strongly correlated with transcription factors other than ARF. For instance, *SmIAA9* was found to cooperate with the *WRKY* (EVM0007532) to regulate stem growth, while *SmIAA22* and the *DBB* (EVM0017797) were observed to antagonize each other and regulate root development. These results suggest that *SmIAAs* perform various unknown functions in different tissues and during different growth and development processes of *S. miltiorrhiza*. The outcomes of this research provide a foundation for further elucidation of the function of the *SmIAAs* and offer valuable resources for improving the characteristics of *S. miltiorrhiza* and breeding new *S. miltiorrhiza* varieties through genetic engineering.

## Supplemental Information

10.7717/peerj.15212/supp-1 Supplemental Information 1 Cis-promoter element of Salvia miltiorrhiza.

10.7717/peerj.15212/supp-2 Supplemental Information 2 IAA protein sequences of all species used to construct the evolutionary tree.

10.7717/peerj.15212/supp-3 Supplemental Information 3 TPM data of all Salvia miltiorrhiza tissues.

10.7717/peerj.15212/supp-4 Supplemental Information 4 Primers for qPCR in Salvia miltiorrhiza.

10.7717/peerj.15212/supp-5 Supplemental Information 5 QPCR’s raw analysis data.

10.7717/peerj.15212/supp-6 Supplemental Information 6 All transcription factors identified in Salvia miltiorrhiza genome.

10.7717/peerj.15212/supp-7 Supplemental Information 7 Each module of the coexpression gene network and its genes.

10.7717/peerj.15212/supp-8 Supplemental Information 8 GO enrichment results of 10 modules.

10.7717/peerj.15212/supp-9 Supplemental Information 9 Evolutionary tree constructed by NJ method.

10.7717/peerj.15212/supp-10 Supplemental Information 10 Chromosome Distribution of IAA in Salvia miltiorrhiza.

10.7717/peerj.15212/supp-11 Supplemental Information 11 Motif logo of IAA protein sequence of Salvia miltiorrhiza.

10.7717/peerj.15212/supp-12 Supplemental Information 12 Phylogenetic trees of three species constructed based on ML method.

## Additional Information and Declarations

### Data Availability

The following information was supplied regarding data availability: The original q-PCR data is available in the Supplemental File.
