# Supplementary figures and images for: Genome-wide identification and co-expression network analysis of Aux/IAA gene family in Salvia miltiorrhiza
Source: PeerJ. 2023 Apr 17;11:e15212. doi: 10.7717/peerj.15212 (PMC10117383; doi:10.7717/peerj.15212)

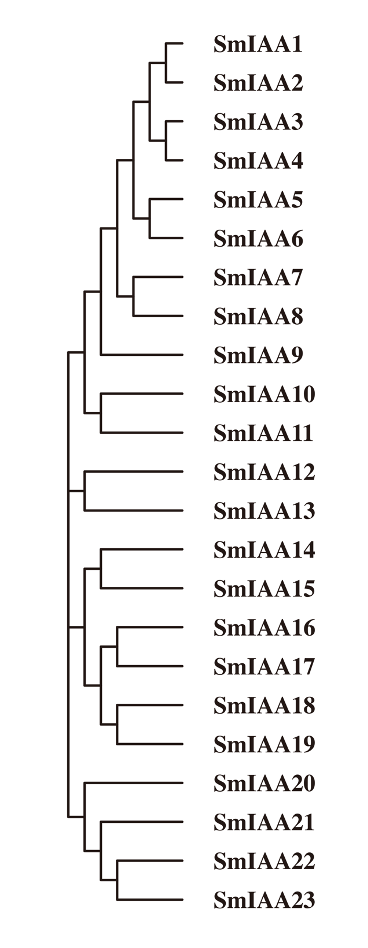

Supplement: Supplemental Information 9 [file peerj-11-15212-s009.png]

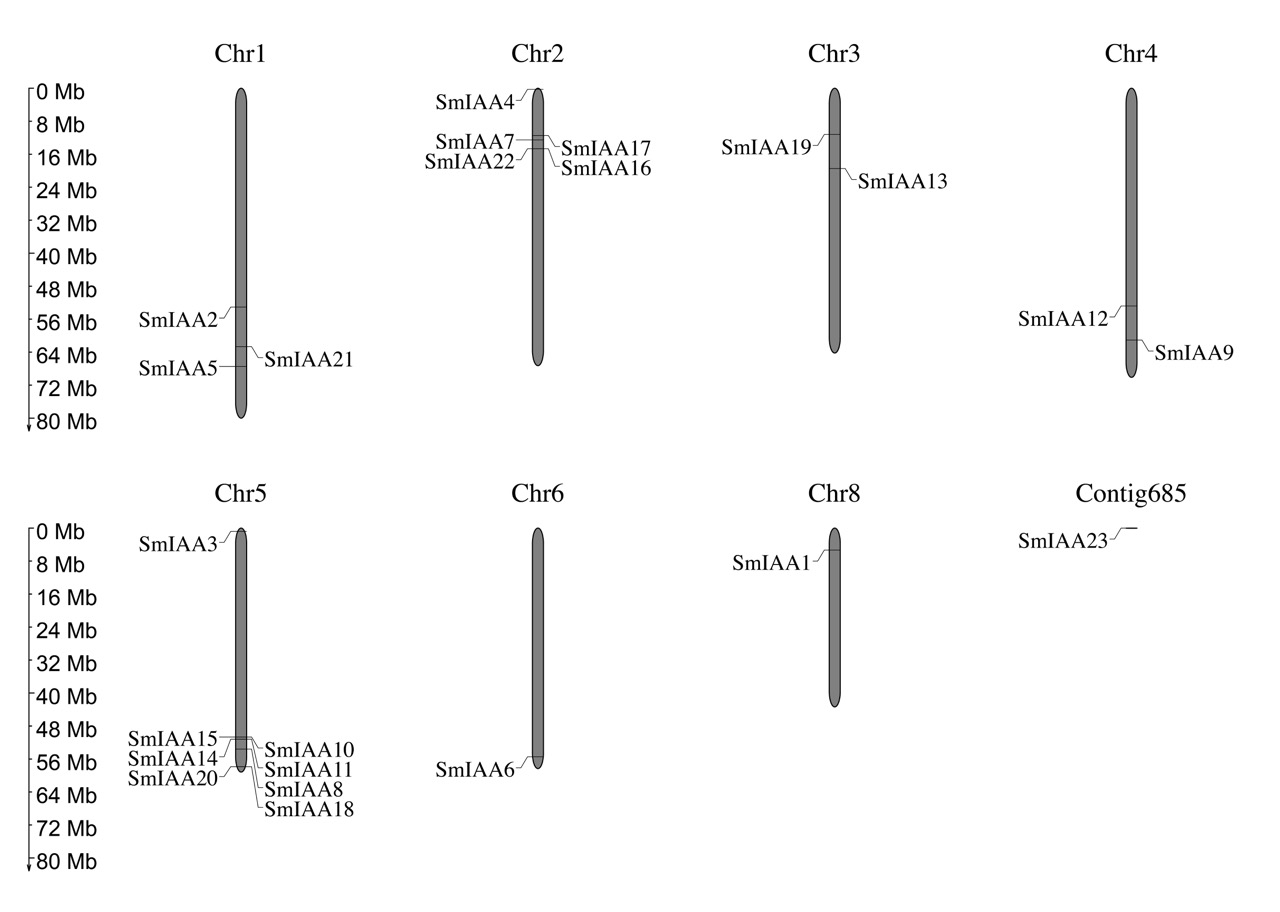

Supplement: Supplemental Information 10 [file peerj-11-15212-s010.jpeg]

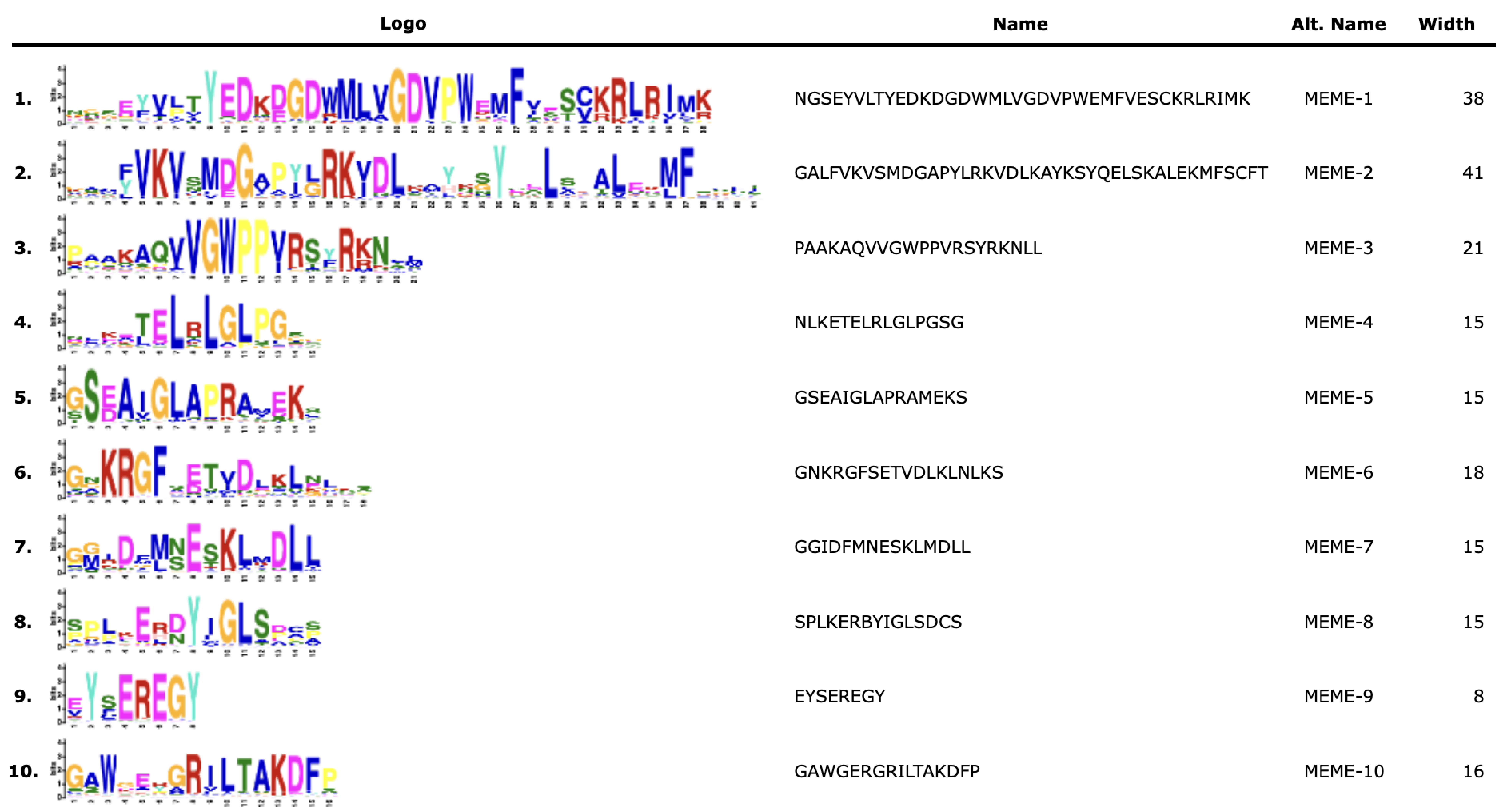

Supplement: Supplemental Information 11 [file peerj-11-15212-s011.png]

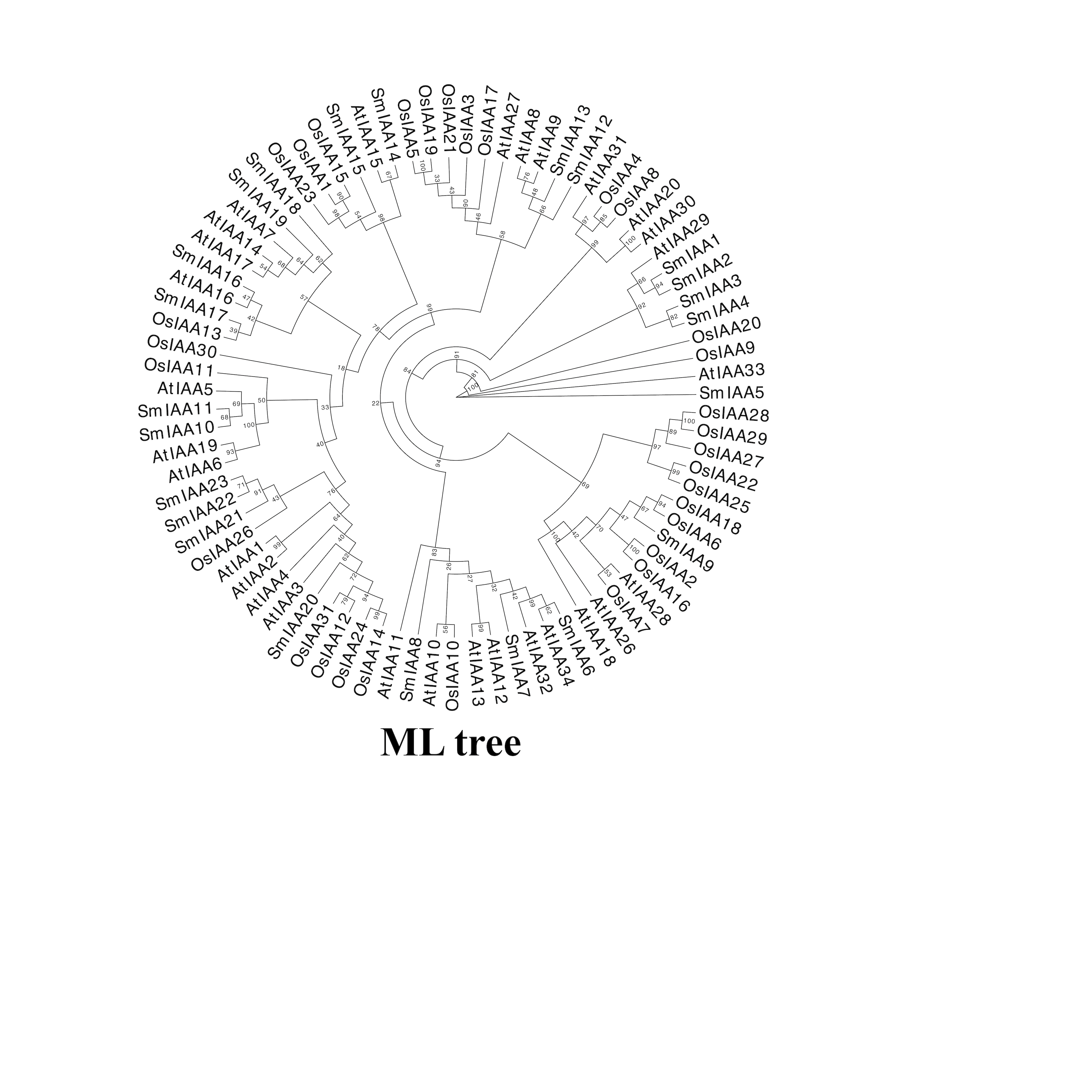

Supplement: Supplemental Information 12 [file peerj-11-15212-s012.png]
